# ASO Author Reflections: RUNX3 in Non-Small Cell Lung Cancer Tumor Microenvironment: Bridging Mechanisms and Clinical Potential

**DOI:** 10.1245/s10434-025-18187-8

**Published:** 2025-09-08

**Authors:** Weijin Xiao, Xiaohui Chen

**Affiliations:** 1 Department of Thoracic Surgery, Clinical Oncology School of Fujian Medical University, Fujian Cancer Hospital, Fuzhou, People’s Republic of China; 2 Interdisciplinary Institute of Medical Engineering of Fuzhou University, Fuzhou, People’s Republic of China

## Past: Unanswered Questions About RUNX3 in Non-Small Cell Lung Cancer

RUNX3 has long been recognized as a tumor suppressor gene (TSG) in various cancers, including non-small cell lung cancer (NSCLC), but its specific biological mechanisms, particularly how it influences the tumor microenvironment (TME), remain poorly understood.^1^ Clinically, NSCLC remains a leading cause of cancer mortality, with its progression closely tied to TME dynamics, including immune cell infiltration and stromal interactions.^2^ Key questions persisted: How does RUNX3 expression status shape the TME in NSCLC? Which cell populations mediate its effects? Could these insights inform therapeutic strategies? Our study aimed to address these gaps using single-cell RNA sequencing (scRNA-seq) to dissect TME changes associated with RUNX3 expression.

## Present: Key Findings and Their Relevance

Our scRNA-seq analysis revealed that RUNX3 expression status is tightly linked to TME composition in NSCLC.^3^ Specifically, RUNX3-positive (RUNX3_Pos) tumors were enriched in fibroblasts, while RUNX3-negative (RUNX3_Neg) tumors accumulated diverse immune cells, including B cells, neutrophils, and plasmacytoid dendritic cells. Critically, mononuclear phagocytes (MPs), encompassing macrophages, monocytes, and dendritic cells, emerged as central players: RUNX3_Pos tumors showed macrophage enrichment, whereas RUNX3_Neg tumors exhibited macrophage depletion and monocyte enrichment.^3^

These findings are significant because they identify MPs as a potential target of RUNX3-mediated TME remodeling. This aligns with broader evidence that tumor-associated myeloid cells (TAMCs) regulate immune responses and tumor progression.^4^ Our data also highlight the role of RUNX3 in modulating antigen presentation pathways (e.g., major histocompatibility class [MHC] class II molecules) and chemotactic signals, offering a mechanistic basis for its tumor-suppressive effects. Together, these results bridge the gap between RUNX3’s TSG function and TME dynamics, providing a framework to explore novel immunotherapeutic strategies.

## Future: Directions for Research

Despite these insights, further work is needed. First, in vivo studies using animal models are required to validate the role of RUNX3 in regulating MPs and TME remodeling. Second, larger cohort studies should explore how RUNX3 expression intensity (beyond binary positivity/negativity) influences TME heterogeneity and clinical outcomes. Third, combining RUNX3 targeting with immunotherapies (e.g., checkpoint inhibitors) warrants investigation, given the association of RUNX3 with antigen presentation pathways. Finally, epigenetic regulation of RUNX3 (e.g., methylation) and its impact on TME should be explored to identify potential therapeutic vulnerabilities.^5^
